# The Main Factors of Induced Demand for Medicine Prescription: A Qualitative Study

**Published:** 2019

**Authors:** Azam Mohamadloo, Saeed Zarein-Dolab, Ali Ramezankhani, Jamshid Salamzadeh

**Affiliations:** a *School of Public Health, Shahid Beheshti University of Medical Science, Tehran, Iran.*; b *School of Medicine, Shahid Beheshti University of Medical Science, Tehran, Iran.*; c *School of Pharmacy, Shahid Beheshti University of Medical Sciences, Tehran, Iran.*

**Keywords:** Patients, Physicians, Prescriptions, Qualitative research, Health services, Medicine

## Abstract

Inappropriate request for health care services which are considered to be unnecessary for the patients has long been addressed by several writers**.** The hypothesis supplier induced demand refers to the induced demand initiated by the supplier who acts in his own economic self-interest rather than patient best interest. The purpose of the present qualitative study was to explore about induced demand and the relevant motivating factors associated with unnecessary prescriptions of medicine. In-depth interviews were used for data generation with a purposive sample of 20 participants who were selected according to their experience. The interviews were transcribed and analyzed. The key themes were identified, named and coded with a sample of quotation. In general, 24 sub-themes or factors were identified and classified into personal, community and institutional themes. Some important factors are asymmetric information, patient expectation, patient poor health literacy, physician›s inadequate knowledge in medicine, neglecting patient rights, financial incentives, barriers in health insurance companies, reimbursement mechanism, marketing and advertising by pharmaceutical companies, Poor financial condition of pharmacies and social interactions. Our results showed that the induced demand for medicine is multifactorial in a health system. Addressing these factors could lead to decrease unnecessary prescription of medicine by a multi-faceted strategy, including curriculum revision, health promotion, and policy making.

## Introduction

Inappropriate request for health care services which are considered to be unnecessary for the patients has long been addressed by several writers (1-3). The hypothesis of supplier-induced demand refers to the induced demand created by health care providers who acts in his own economic self- interest rather than patient best interest (3-6). The requested inappropriate health care services include a variety of medical interventions ranging from simple drug prescriptions to complicated surgical interventions, imposing an additional burden to community health care system (7, 8). 

The major motivating factor involved in creating induced demand is reported to be as financial incentives (8-12). Reynolds and McKee in a study conducted on factors inﬂuencing prescribing antibiotics in China found out that clinicians overprescribed antibiotics because they shared in the profit made by pharmaceutical suppliers and hospitals (9). While certain studies indicate that asymmetric information between physician and patient contributes to the creation of induced demand (6, 13-16), Shih and Tie-Seale reported a positive correlation between patient medical information and physicians frequency of prescriptions based on patient-initiated induced demand (3), in that, the patients who had more knowledge regarding their condition and the relevant medication asked for more medical prescriptions. 

One of the conditions in which induced demand is reported to be higher is where the physicians are paid for the services they provide for the patients. Thus, fee for service physicians is regarded to create more induced demands (16-18). Devlin and Sarma reported that fee-for-service physicians had a higher number of patient visits as compared with non-fee for service physicians (18). However, a few reports indicated that there are no differences in the provision of health care services between fee-for-service and non-fee for service physicians. Grytten and Sorensen conducted a study to compare two groups of physicians in Norway, with and without financial motivations to create induced demand, reported that there was no difference in the mean number of laboratory tests per consultation between salaried and contract physicians (19) providing no evidence for physician- induced demand hypothesis. 

Patient-initiated induced demand is another motivating factor for the generation of induced demand (16-18). Schommer et al, in a study conducted on patient-initiated induced demand, found out that patients who requested certain prescriptions had a closer relation with physician and felt more satisfied with their treatment (20). Brekke and Kuhn argue that drug-advertisement may encourage people to seek unnecessary medical advice, and pressurize the physician to prescribe unnecessary medication (21). Cockburn J and Pit S. in a study conducted on prescribing behavior found out that, when the general practitioner knew that the patient expected medication, the possibility of providing the patient with an unnecessary prescription was 10 times more than when the physician thought that the patient did not expect any medication (22). However, reports indicate that the patients may not pressurize the physician for prescription but indulge in self-medication. Eng et al demonstrated that patients take antibiotics for common cold as self-medication (23).

In the Iranian health system under the third reform called the Health Sector Evolution Plan, launched in 2014, physicians work in the hospitals operate under both fee for service and non-fee for service program and they have both a fixed monthly payment and a per case payment (24). Thus, the purpose of the present qualitative study was to reconsider induced demand and investigate the motivating factors for unnecessary prescription of medicine in the Iranian context through in-depth interviews with faculty members, physicians, pharmaceutics, and patients. 

## Experimental

The induced demand is regarded as a negative behavior, and considering just the experiences of physicians may lead to underreporting and consequently missing valuable information. Therefore, a qualitative study was designed and conducted on a purposive sample of 20 various stakeholders: 12 faculty members and 8 non faculty members. Of 12 faculty members, 3 were health educationists, 5 health economics, 1 clinical pharmacologist, 2 pharmacologists, 1 pharmaco-economist; and of 8 non faculty members, 2 were pharmaceutics, 4 General Practitioners (GPs) and 2 patients as consumers. All participants wer'e over the age of 37. Except the two patients, all had more than 10-year experiences in their own fields. To explore the participants' experiences and opinions about induced demand for medicine prescription, 20 in-depth interviews were held from September to December of 2015 in Tehran.

After receiving informed consent and permission for voice recording, all participants were interviewed by the author A.M who had formal education in interviewing. The participants were reassured of maintaining respondent confidentiality and anonymity. Each interview was exploratory in nature and lasted for 30-60 min. The participants were asked about their experiences and views about induced demand for medicine prescription. New questions were added or refined as the interview process progressed. All the questions were open. The interviews were stopped, transcribed, and analyzed when the data were saturated. The key themes were identified, named, and coded with at least one sample of quotation. To avoid bias and receive consensus, all authors participated in the analysis process. To increase the validity and trustworthiness of the analysis, the themes, codes, and quotations were double checked with the participants who were faculty members to get consensus over any interpretations. The interview method and the analyses were also double-checked and verified by two experts (faculty members) in the field of qualitative research. 

## Results

In the process of data analysis, all the factors associated with induced demand for prescription of medicine were elicited from the data analysis and were classified into three themes: personal (intra and inter personal), community, and institutional. The themes were subdivided into 24 sub themes or factors (Table1), in that, 6 were personal, 8 community and 10 institutional.

*Personal factors of induced demand for medical prescription*

The interviews revealed six personal factors leading to unnecessary prescription of medicine. The personal factors are defined in terms of knowledge, attitude, beliefs, personality traits, and social relationships (25). These factors are mainly related to physician and patient including asymmetric information, physician’s inadequate knowledge in medicine, inadequate knowledge in health economy, patient expectation for specific medicine prescription, patient poor health literacy, and social interactions.

Asymmetric information*:* Asymmetric information refers to inadequate knowledge of the patient regarding his condition. Of 20 interviews, 10 confirmed the relation between asymmetric information and creation of induced demand. Two samples are mentioned here: "One of the major problems in our health economy is that most patients don’t know what they want. You may go shopping and know what you want, know the brand you want, length and price, but in the health system, I will be very clear saying that patients do have none of this information and we never provide this information to patients" (a health educationists, P 19). "The cause here is asymmetric information between the provider that could be a physician or pharmacist and the recipient of services that can be a consumer or client" (a pharmaco-economist, Interview No. 9

Physicians’ inadequate knowledge of medicine*:* Of 20 interviews, 11 confirmed that physician’s inadequate knowledge of medicine inadvertently created induced demand. Two samples are mentioned here: "Misdiagnosis and efficient knowledge of some physicians consciously lead them towards inducing demand for medical prescription in patients. That means they are not very expert in their practice" (a Pharmaco-economist, P 9). "If a doctor had enough information, he would not have prescribed that" (a health economist, P 5).

Inadequate knowledge in health economy*:* Five interviews confirmed that most of the physicians had poor knowledge in health economy that inadvertently led to induced demand. One sample of the interview is presented here: "I think they can be informed on this issue because most physicians have not taken a course on issues in economics or management and they are not willing to get involved in them" (a health economist, P 7).

Patient’s expectation for specific medicine prescription*:* All interviews confirmed the patient’s expectation for specific medicine prescription. Three interviews are presented here: "Most patients expect the physician to prescribe a large sum of drugs, thus a good physician is regarded as the one who prescribes more medicine specially, intravenous ones" (a GP, P 18). "A patient may not even request antibiotics such as Cefixime, but I’ll prescribe it because we can’t tolerate anymore; you know, in many instances we prescribe something before the patient requests for that, we are really fed up with inappropriate patients’ demands, we know what they expect from us " (a GP, P 17). "It may be the patient who demands from physician to prescribe a specific drug and physicians sometimes give in" (a GP, P 16).

Patient poor health literacy: Patient poor health literacy is defined as inadequate knowledge of medicine and in general low health literacy. Out of 16 interviews stating patient poor health literacy, one sample is: "Yesterday a patient said that he took an Adult Cold, acetaminophen codeine, and amoxicillin, and then I asked him how many days he had taken the antibiotics? The patient said that he had taken the medicine for 2 days and he got fine, obviously the patient did not know that he should have taken the antibiotics at least for three days. Thus, if he got fine it is because he did not need the antibiotics. (a pharmacist, P 11).

**Table 1:** Factors associated with induced demand for prescription of medicine

**Theme**	**Sub-Theme (Factors)**
Personal factors	Asymmetric information
	physician's inadequate knowledge in medicine
	Inadequate knowledge in health economy
	Patient expectation for specific medicine prescription
	Patient poor health literacy
	Social interactions
Community factors	Neglecting patient rights
	Financial incentives
	Trafficking medicine
	Marketing and advertising by pharmaceutical companies
	Poor financial condition of pharmacies
	Excessive trust in physicians
	Patient’s satisfaction
	Affordability of the patient
Institutional factors	Inefficiency of health insurance companies
	Barriers in the development and implementation of drug regulations
	Barriers in production, distribution and supply of health resources
	Weakness in monitoring and controlling the health system
	Overemphasis of treatment-based health system
	Weakness in the structure of the health system
	Inadequate emphasis on the role of medicine in curriculum
	Inadequate knowledge in health information technology
	Weakness in the drug pricing system
	Uncontrolled reimbursement mechanism in health system

*Community factors of induced demand for medical prescription*

The interviews revealed 8 community factors leading to unnecessary prescription of medicine. The community factors are defined in terms of social networks, norms, or standards among individuals, groups, and organizations (25). Factors were as follows: neglecting patient rights, financial incentives, trafficking medicine, marketing and advertising by pharmaceutical companies, Poor financial condition of pharmacies, excessive trust in physicians, patient’s satisfaction, and affordability of the patient. Two of the factors with samples of interviews are mentioned below. 

Neglecting patient rights*:* The factors affecting patient’s rights are defined as unclear complaint system, patient’s unawareness of his rights, lack of a system for the provision of medical information to patients, poor communication between physician and patient.

Two out of 12 quotes reflecting the reasons for neglecting the patient rights are as follows: "patients are uninformed of their rights and abuse comes from this unawareness" (a GP, P 20), "Some physicians want to have a short visit with the patient because, their office is busy; so they don’t spend adequate time to educate, explain, and mention the complications of drugs" (a GP, P 16).

Financial incentives*:* Financial incentive is regarded as one of the main factors in creating induced demand for medical prescriptions. There are usually three stakeholders involved: physicians, pharmacists, and pharmaceutical companies. Out of 18 interviews stating the financial incentives, three interview samples are reported. "They do over prescription since they want to have more income" (a GP, P 20). "The greatest work of the companies is lobbying with individuals and organizations, individuals might be physicians and clinics, and organization might be hospital that they somehow claw each other they say you send the patient here to get these drugs and we will send patients to you in exchange" (a health educationist, P19). A drug store rang me and said, could you prescribe these medicines because they are close to expiry dates. (a GP, 20) 

*Institutional factors of induced demand for medical prescription*

The interviews revealed 10 institutional factors leading to the unnecessary prescription of medicine. The institutional factors are defined in terms of rules, regulations, policies, and informal structures, which may constrain or promote recommended behaviors (25). Factors were as follows: inefficiency of health insurance companies, barriers in the development and implementation of drug regulations, barriers in production, distribution and supply of health resources, weakness in monitoring and controlling health system, overemphasis of treatment-based health system, weakness in the structure of the health system, inadequate emphasis on the role of medicine in curriculum, inadequate knowledge in health information technology, weakness in the drug pricing system, and uncontrolled reimbursement mechanism in health system.

Inefficiency of health insurance companies is defined as parallel health insurance companies, inadequate supervision in health insurance companies and inefficient rules and regulations of health insurance companies. Out of 16 interviews stating inefficiency of health insurance companies, two samples are: "Our other problem is that insurances are not integrated together and they work separately and do not exchange information with other insurance companies. In hospitals, social security insurance has its own operating system, while health care insurance has a different system" (a health educationist, P 19). "Because insurance companies do not monitor the prescription of drugs, thus the physicians do not care about their prescriptions "(a GP, P 17).

Barriers in the development and implementation of drug regulations refer to softness in the performance bond, inefficiency in executing the rules, ignoring the law of not selling the prescription medicines on-the-counter. Eighteen interviews confirmed inefficiency in the development and implementation of drug regulations. One sample of the interview is: "pharmacists know that there is no punishment for selling antibiotics to patients without a prescription, and they sell it with no hesitation" (a GP, P 20).

Barriers in production, distribution, and supply of health resources are defined as ineffective ‶drug supply management, drug unavailability, and barriers in the distribution and training manpower resources. Six interviews confirmed barriers in production, distribution, and supply of health resources. Three samples of the interviews are: "the patient buys a lot of drugs that he needs in the near future because he is not certain that later the drug can be found, thus drug supply instability caused the patient to create drug induced demand either by asking the physician to prescribe certain medicines or by asking the pharmacist to sell the drug on the counter" (a GP, P. 20). "When we cannot provide a drug regularly according to schedule, uncertainty is created" (a pharmacologist, P 12). "In many big cities in our country, like Shiraz, there are a lot of physicians all specialists or sub-specialists just on Zand street, but when you go to small cities such physicians are very scarce" (a health economist, P 7). 

Weakness in monitoring and controlling the health system was confirmed by 12 interviews, one sample of the interview is presented here: "First we must know and accept that our system is an ill system and then think about how to treat this ineffective system" (a pharmacologist, P 12).

Weakness in the structure of the health system is defined as disintegration of the health system, Weakness in the interrelationship between medical professionals, lack of clinical guidelines. Out of 14 interviews confirming the weakness in the structure of the health system, two samples of the interviews are presented here: "If we don’t have a systematic, coherent, structured systems, induced demand may be created; when a person knows that there is no supervision, and he has no commitment to his own profession, we have created the condition for this person to take advantage of the system in any possible form such as induction" (a health educationist, P 19). "Due to a complete weakness in booking visits with physicians and in general weakness in communications in the health system, there is more burden on paraclinical therapies" (a pharmacologists, P 13(. 

Weakness in the medical curriculum in that the physicians are trained with the emphasis on prescription of medicine, rather than on prevention or management strategies to avoid unnecessary prescritions. This was stated in two interviews. One sample of the interviews is "educational system trains physicians in a way that their emphasis is more on the medicine" (a health economics, P 10).

## Discussion

According to the results of this qualitative analysis, many factors were reported to be involved in inducing unnecessary demand, which were divided into three categories personal, community, and institutional factors. This implies that health system is a multifactorial one that one aspect of the medical health profession is interrelated with other aspects and cannot be considered separately. The unnecessary prescription of medicine was influenced by many different factors from a simple personal factor to more complicated community and institutional factors in a particular health system. While most studies look at induced demand as the physician motives for self-interest (6, 13-15), the present study showed that there are many other interrelated factors involved in this multifactorial process. 

The personal factors reported in the current study provide more evidence for the studies suggesting the following factors for creating induced demand for medical prescriptions: asymmetric information (4, 16, 26), physician’s inadequate knowledge in medicine (27-30), patient expectation for specific drug prescription (30-33), and patient poor health literacy (34, 35). A study conducted on exploring obstacles to appropriate practice of physician showed that a gap in clinical knowledge and also being unawareness of the patient’s condition are obstacles for rational prescription (28). Akkerman *et al*., in a study conducted on exploring determinants of over-prescribing antibiotics, reported that overestimating symptoms by physicians and also patients’ expectations could be determinants of unnecessary prescription of medicine (30). While the results of the present study provide more evidence for Akkerman *et al*. and similar studies, we report social interactions and physician’s inadequate knowledge of health economy as another factors which may lead to creating induced demand. 

The community factors reported in our study provide more evidences for the studies suggesting the following factors important in creating induced demand for medical prescriptions: marketing and advertising by pharmaceutical companies (21, 36-39), excessive trust to the physician (33, 40), financial incentives (8-12), and affordability of the patient (41). Frosch *et al*., in a study exploring the impact of medicine advertisement on creating demand for medication, reported that medicine advertisement possibly provoked patients to demand inappropriate prescriptions. Since most advertisements have limited educational value and in many cases provide inadequate information about a medical condition, they may mislead the patient in believing that they are useful for his condition (37). While our results are in agreement with those of Frosch *et al*. study, we report more community factors involved in creating induced demand such as neglecting patient’s rights, trafficking medicine, poor financial condition of pharmacies, and patient satisfaction which have not been reported in the previous studies because most studies have only focused on the physicians and patients. 

The institutional factors reported in our study provide more evidence for the studies suggesting factors of induced demand for medical prescriptions such as inefficiency of health insurance companies (42-44), weakness in monitoring and controlling the health system (26, 4), weakness in the structure of the health system (45), and also uncontrolled reimbursement approach in health system (7, 16, 18). Madden *et al*., in a study conducted on impact of the reimbursement system on GPs’ behavior, reported that health systems with fee-for-service reimbursement method contributed to creating induced demand (7). However, in addition to the institutional factors reported in the previous studies, the results of the current study indicate other factors involved in creating induced demand for medical prescription such as: barriers in the development and implementation of drug regulations; barriers in production, distribution, and supply of health resources; overemphasis of treatment-based health system; inadequate knowledge in health information technology; weakness in the drug pricing system. These factors have not been reported in the previous studies. One major reason of this difference is that in our qualitative study, all the questions were open and directed toward expert people experienced in various fields of the health system. 

One of the limitations of the study was that we might have missed some information because we could not extend the time of the interviews due to the limited time our participants had. However, we have well managed the interview to get the best out of that by asking the right questions and letting the participants inform us of what they knew through open ended questions. 

To conclude, the present study provides Iranian evidence for confirming the induced demand hypothesis and demonstrates that supplier induced demand hypothesis is multifactorial in nature and various aspects of the health system need to be overviewed and reconsidered in order to reduce unnecessary prescriptions of medicine. 

We recommend further studies exploring induced demand in surgeries, particularly aesthetic reconstructive surgeries and unnecessary caesarians. 

## References

[1] McGuire TG (2000). Physician agency. Handbook of health economics.

[2] Guire TG (2000). Physician agency. Handbook of health economics.

[3] Carlsen F, Grytten J (2000). Consumer satisfaction and supplier induced demand. J. Health Econ.

[4] Shih YCT, Tai-Seale M (2012). Physicians' perception of demand‐induced supply in the information age: a latent class model analysis. Health Econ.

[5] Blomqvist Å (1991). The doctor as double agent: Information asymmetry, health insurance, and medical care. J. Health Econ.

[6] Labelle R, Stoddart G, Rice T (1994). A re-examination of the meaning and importance of supplier-induced demand. J. Health Econ.

[7] J Sørensen R, Grytten J (1999). Competition and supplier‐induced demand in a health care system with fixed fees. Health Econ.

[8] Madden D, Nolan A, Nolan B (2005). GP reimbursement and visiting behaviour in Ireland. Health Econ.

[9] Clemens J, Gottlieb JD (2014). Do physicians' financial incentives affect medical treatment and patient health? Am. Econ. Rev.

[10] Reynolds L, McKee M (2009). Factors influencing antibiotic prescribing in China: an exploratory analysis. Health Policy.

[11] Kotwani A, Wattal C, Katewa Sh, Joshi PC, Holloway K (2010). Antibiotic use in the community: what factors influence primary care physicians to prescribe antibiotics in Delhi, India?. Fam. Pract.

[12] Mao W, Tang S, Chen W (2013). Does perverse economic incentive lead to the irrational uses of medicines?. Expert Rev Pharmacoecon Outcomes Res.

[13] Chen M, Wang L, Chen W, Zhang L, Jiang H, Mao W (2014). Does economic incentive matter for rational use of medicine? China’s experience from the essential medicines program. Pharmaco. Economics.

[14] Delattre E, Dormont B (2003). Fixed fees and physician‐induced demand: A panel data study on French physicians. Health Econ.

[15] Rice TH, Labelle RJ (1989). Do physicians induce demand for medical services? J. Health Polit. Policy Law.

[16] Peacock SJ, Richardson JR (2007). Supplier-induced demand: re-examining identification and misspecification in cross-sectional analysis. Eur. J. Health Econ.

[17] Blomqvist Å, Léger PT (2005). Information asymmetry, insurance, and the decision to hospitalize. J. Health Econ.

[18] Feldman R, Sloan F (1988). Competition among physicians, revisited. J. Health Polit. Policy Law.

[19] Devlin RA, Sarma S (2008). Do physician remuneration schemes matter? The case of Canadian family physicians. J. Health Econ.

[20] Grytten J, Sorensen R (2001). Type of contract and supplier-induced demand for primary physicians in Norway. J. Health Econ.

[21] Schommer JC, Singh RL, Hansen RA (2005). Distinguishing characteristics of patients who seek more information or request a prescription in response to direct-to-consumer advertisements. RSAP.

[22] Brekke KR, Kuhn M (2006). Direct to consumer advertising in pharmaceutical markets. J. Health Econ.

[23] Cockburn J, Pit S (1997). Prescribing behaviour in clinical practice: patients' expectations and doctors' perceptions of patients' expectations—a questionnaire study. BMJ.

[24] Eng JV, Marcus R, Hadler JL, Imhoff B, Vugia DJ, Cieslak PR, Zell E, Deneen V, McCombs KG, Zansky SM, Hawkins MA (2003). Consumer attitudes and use of antibiotics. Emerg. Infect. Dis.

[25] Moradi-Lakeh M, Vosoogh-Moghaddam A (2015). Health sector evolution plan in Iran; equity and sustainability concerns. Int. J. Health Policy Manag.

[26] Glanz K, Rimer BK, Viswanath K (2008). Health behavior and health education: theory, research, and practice.

[27] Bickerdyke I, Dolamore R, Monday I, Preston R (2002). Supplier-induced demand for medical services. Canberra: Productivity Commission Staff Working Paper.

[28] Britten N, Stevenson FA, Barry CA, Barber N, Bradley CP (2000). Misunderstandings in prescribing decisions in general practice: qualitative study. BMJ.

[29] Ely JW, Osheroff JA, Ebell MH, Chambliss ML, Vinson DC, Stevermer JJ, Pifer EA (2002). Obstacles to answering doctors' questions about patient care with evidence: qualitative study. BMJ.

[30] Yousefi N, Majdzadeh R, Valadkhani M, Nedjat S, Mohammadi H (2012). Reasons for physicians’ tendency to irrational prescription of corticosteroids. Iran. Red Crescent Med. J.

[31] Akkerman AE, Kuyvenhoven MM, van der Wouden JC, Verheij TJ (2005). Determinants of antibiotic overprescribing in respiratory tract infections in general practice. J. Antimicrob. Chemother.

[32] Britten N, Ukoumunne O (1997). The influence of patients' hopes of receiving a prescription on doctors' perceptions and the decision to prescribe: a questionnaire survey. BMJ.

[33] Coenen S, Michiels B, Renard D, Denekens J, Van Royen P (2006). Antibiotic prescribing for acute cough: the effect of perceived patient demand. Br. J.Gen. Pract.

[34] Khorasani E, Keyvanara M, Karimi S, Jafarian Jazi M (2014). The Role of Patients in Induced Demand from Experts’ Perception: A Qualitative Study. Journal of Qualitative Research in Health Sciences.

[35] Otoom S, Sequeira R (2006). Health care providers' perceptions of the problems and causes of irrational use of drugs in two Middle East countries. Int. J. Clin. Pract.

[36] Currie J, Lin W, Zhang W (2011). Patient knowledge and antibiotic abuse: Evidence from an audit study in China. J. Health Econ.

[37] Gilbody S, Wilson P, Watt I (2005). Benefits and harms of direct to consumer advertising: a systematic review. BMJQual Saf.

[38] Frosch DL, Krueger PM, Hornik RC, Cronholm PF, Barg FK (2007). Creating demand for prescription drugs: a content analysis of television direct-to-consumer advertising. Ann. Fam. Med.

[39] Gellad ZF, Lyles KW (2007). Direct-to-consumer advertising of pharmaceuticals. Am. J. Med.

[40] Mintzes B, Barer ML, Kravitz RL, Bassett K, Lexchin J, Kazanjian A, Evans RG, Pan R, Marion SA (2003). How does direct-to-consumer advertising (DTCA) affect prescribing? A survey in primary care environments with and without legal DTCA. Can. Med. Assoc. J.

[41] Thom DH, Kravitz RL, Bell RA, Krupat E, Azari R (2002). Patient trust in the physician: relationship to patient requests. Fam. Pract.

[42] Doran E, Robertson J, Henry D (2005). Moral hazard and prescription medicine use in Australia—the patient perspective. Soc. Sci. Med.

[43] Soofi M, Bazyar M, Rashidian A (2011). Types of moral hazards and its effects on insurance marketing and health system. Hospital.

[44] Dusansky R, Koç Ç (2010). Implications of the interaction between insurance choice and medical care demand. J. Risk Insur.

[45] Winkelmann R (2004). Co-payments for prescription drugs and the demand for doctor visits–Evidence from a natural experiment. Health Econ.

[46] Khorasani E, Keyvanara M, Karimi S, Jazi MJ (2014). Views of health system experts on macro factors of induced demand. Int. J. Prev. Med.

